# Efficacy of antioxidant intervention and exercise intervention for lipid peroxidation in dialysis patients: a meta-analysis

**DOI:** 10.3389/fmed.2025.1473818

**Published:** 2025-03-17

**Authors:** Mengyuan Yu, Shengmao Liu, Jian Li, Ce Ni, Xinyang Li, Wenpeng Cui

**Affiliations:** Department of Nephrology, The Second Hospital of Jilin University, Changchun, China

**Keywords:** lipid peroxidation, hemodialysis, peritoneal dialysis, antioxidant intervention, exercise intervention

## Abstract

**Background:**

Lipid peroxidation is a major factor known to contribute to occurrence of cardiovascular events in dialysis patients. This study aims to investigate whether antioxidant interventions can improve lipid peroxidation damage in dialysis patients.

**Methods:**

A comprehensive search in PubMed, Embase, and the Cochrane Library was conducted to identify eligible randomized controlled trials (RCTs) up to June 2024. Endpoints of interest included biomarkers related to Lipid peroxidation. The results from eligible studies were performed using RevMan 5.3 and Stata17.0 software.

**Results:**

A total of 25 RCTs were included, involving eight interventions such as vitamin C supplementation, vitamin E supplementation, vitamin E-coated dialyzer, ω-fatty acid supplementation, curcumin supplementation, pomegranate juice supplementation, exercise intervention, and multiple antioxidant interventions. Outcome indicators included malondialdehyde (MDA) and oxidized low-density lipoprotein (Ox-LDL). The meta-analysis revealed that vitamin E supplementation caused significant reductions in MDA (*p* = 0.01). Treatment with vitamin E-coated dialyzer markedly decreased MDA levels (*p* < 0.0001). Curcumin supplementation significantly reduced Ox-LDL levels (*p* = 0.03). Exercise intervention decreased MDA levels (*p* < 0.0001). Multiple antioxidant interventions significantly decreased MDA (*p* = 0.01).

**Conclusion:**

Supplementation of vitamin E, vitamin E-coated dialyzer treatment, curcumin supplementation, exercise intervention, and multiple antioxidant interventions can effectively reduce the level of lipid peroxidation biomarkers in dialysis patients.

**Systematic review registration:**

https://www.crd.york.ac.uk/PROSPERO (CRD42023455399).

## 1 Introduction

Oxidative stress (OS) refers to the imbalance between excess oxidants (free radicals) and the complete clearance of these free radicals by the internal antioxidant system (1). The kidneys, due to their abundant polyunsaturated fatty acids, are particularly susceptible to attacks from reactive oxygen species (ROS) (2). Studies have demonstrated that as kidney function deteriorates, OS gradually worsens (3). From 2003 to 2016, while incidence rates of treated end-stage renal disease (ESRD) remained relatively stable in many high-income countries, they significantly increased, particularly in East and Southeast Asia. In 2015, China had an estimated prevalence of 402 individuals per million receiving hemodialysis (HD) and 40 per million receiving peritoneal dialysis (PD), corresponding to approximately 553,000 HD patients and 55,000 PD patients (4). In clinical practice, HD is one of the main renal replacement therapies for ESRD patients. However, during HD, blood remains exposed to the dialyzer membrane or dialysis solution. This non-selective solute removal process results in the loss of essential substances, including antioxidants (5, 6). Meanwhile, the complement factors, platelets, and polymorphonucleus are activated within minutes following HD treatment (7), which exacerbates OS in HD patient. As an alternative therapy for ESRD patients, PD, offers higher biocompatibility than HD. A recent meta-analysis found that diabetic renal failure patients treated with PD had a lower incidence of cardiovascular events than HD (8). Nonetheless, due to the composition of PD fluid, peritoneal cells are exposed to a high-glucose environment caused by glucose or its degradation products, leading to OS damage (9). Overall, dialysis patients experience elevated OS.

Some clinical studies have attempted to develop antioxidant interventions for dialysis patients. However, these studies employ varied antioxidant intervention methods, leading to inconsistent conclusions. For example, vitamin C, a universal antioxidant, has demonstrated efficacy against OS in multiple clinical studies (10–13). However, a clinical trial study by De Vriese et al. (14) suggested that vitamin C supplementation could aggravate OS in dialysis patients. Presently, there is no meta-analysis addressing vitamin E supplementation in HD patients. There was only one meta-analysis on vitamin E supplementation in hemodialysis patients (15). However, the included literature does not consist solely of high-quality RCTs, resulting in high heterogeneity and controversial conclusions. A double-blind controlled experiment by Ahmadi et al. (16) found that vitamin E alone did not significantly alleviate OS in dialysis patients. Yet, the combination of vitamin E and alpha-lipoic acid significantly reduced OS. The use of anti-inflammatory substances in dialysis patients has gained increasing attention. A recent meta-analysis suggests that curcumin-containing supplements may modulate inflammatory biomarkers in HD patients (17). Beyond conventional antioxidants, emerging evidence highlights the therapeutic potential of phytochemical-rich interventions. Notably, pomegranate has demonstrated significant antioxidant efficacy in clinical settings (18). A recent review suggested that pomegranate may exert antioxidant, anti-inflammatory effects and improve blood lipids in hemodialysis patients (19). Consequently, a comprehensive meta-analysis of all available research in this field is imperative.

Oxidative stress is primarily assessed based on oxidation end products, such as lipid peroxidation, DNA damage, and end products of protein and amino acid oxidation. Lipids, as essential components of cell membranes or lipoproteins, are susceptible to ROS attacks due to their active double bonds (20). Lipid peroxidation participates in the development of atherosclerosis, a major contributor to cardiovascular disease (21). Cardiovascular complications represent a significant cause of mortality in patients with ESRD (22). A cohort study utilizing the US Renal Data System revealed that cardiovascular disease accounted for nearly 40% of all deaths in ESRD patients, a rate 500 times higher than that observed in the general population (23). Oxidized low-density lipoprotein (Ox-LDL), an early OS marker of oxidative stress and modified form of LDL, has been extensively studied in HD patients as a potential indicator of atherosclerosis-associated diseases (24). Therefore, lipid peroxides were selected as markers of OS in this meta-analysis. This study aims to consolidate existing evidence regarding the effects of antioxidant intervention on lipid peroxidation in dialysis patients.

## 2 Methods

### 2.1 Protocol registration

The study followed the Preferred Reporting Items for Systematic Reviews and Meta-Analyses (PRISMA) recommendations (25). The review protocol was registered on PROSPERO (CRD42023455399).

### 2.2 Information sources

A search was performed on June 30, 2024 on the PubMed, Embase, and the Cochrane Library to identify eligible studies, specifically focusing on population-based RCTs, without restriction to language preference.

### 2.3 Inclusion criteria

(1) Population-based studies. (2) The study type was RCT. (3) Study participants must be at least 18 years of age. (4) Receiving regular HD (3 times a week) as kidney replacement therapy for ≥2 months. (5) The study reported blood biomarker levels of lipid peroxidation.

### 2.4 Exclusion criteria

(1) Reviews, *in vitro* studies, case reports, conference minutes, and other literature were excluded. (2) Studies involving participants with malignant or acute inflammatory diseases, cancer, or other major underlying diseases were excluded. (3) Articles lacking complete data were excluded. (4) Duplicate data and articles that could not be meta-analyzed were excluded.

### 2.5 Search strategy

Search keywords included terms such as “hemodialysis,” “peritoneal dialysis,” “lipid peroxidation,” “malondialdehyde,” “thiobarbituric acid reactive substances,” “TBARs,” “Ox-LDL,” “8-iso-prostaglandin F_2α_” and “4-hydroxynonenal.” The search strategy involved all possible combinations of subject terms and free words.

### 2.6 Data collection process

The selection process was divided into two steps, each carried out independently by two reviewers. Discrepancies between reviewers were resolved with the assistance of a third researcher.

### 2.7 Study risk of bias assessment

Two reviewers independently assessed study quality using the Cochrane Collaboration’s risk of bias assessment criteria. To minimize bias in evaluation, discrepancies were resolved through discussion and consensus between the two reviewers, ensuring evaluation consistency.

### 2.8 Data extraction

Two researchers independently used standardized tables to extract data including study design, study population, age, dialysis duration, country, biomarker level, sample source, and comparison results. Non-standard data types were transformed for extraction. Outcomes were measured with quantitative biomarkers levels (mean ± standardized mean difference). The data were presented in tabular form.

### 2.9 Synthesis methods

A meta-analysis was performed using RevMan 5.3 and Stata17.0 software, quality assessment was conducted using the Cochrane risk of bias assessment tool. And *I*^2^ statistics were used to assess heterogeneity in the literature (26). An *I*^2^ = 0 indicated no heterogeneity among studies. When the *I*^2^ < 50%, it signified a lack of significant heterogeneity among the studies, and the fixed-effect model was applied. In cases where the *I*^2^ ≥ 50%, indicating significant heterogeneity, the random effects model was applied. The source of heterogeneity was explored through meta-regression and subgroup analysis (27). Sensitivity analysis was conducted for outcome indicators with more than three studies. Egger regression test and funnel plots were employed to investigate the level of publication bias among the outcome indicators with more than two studies (28).

## 3 Result

### 3.1 Study selection

**Figure 1:**
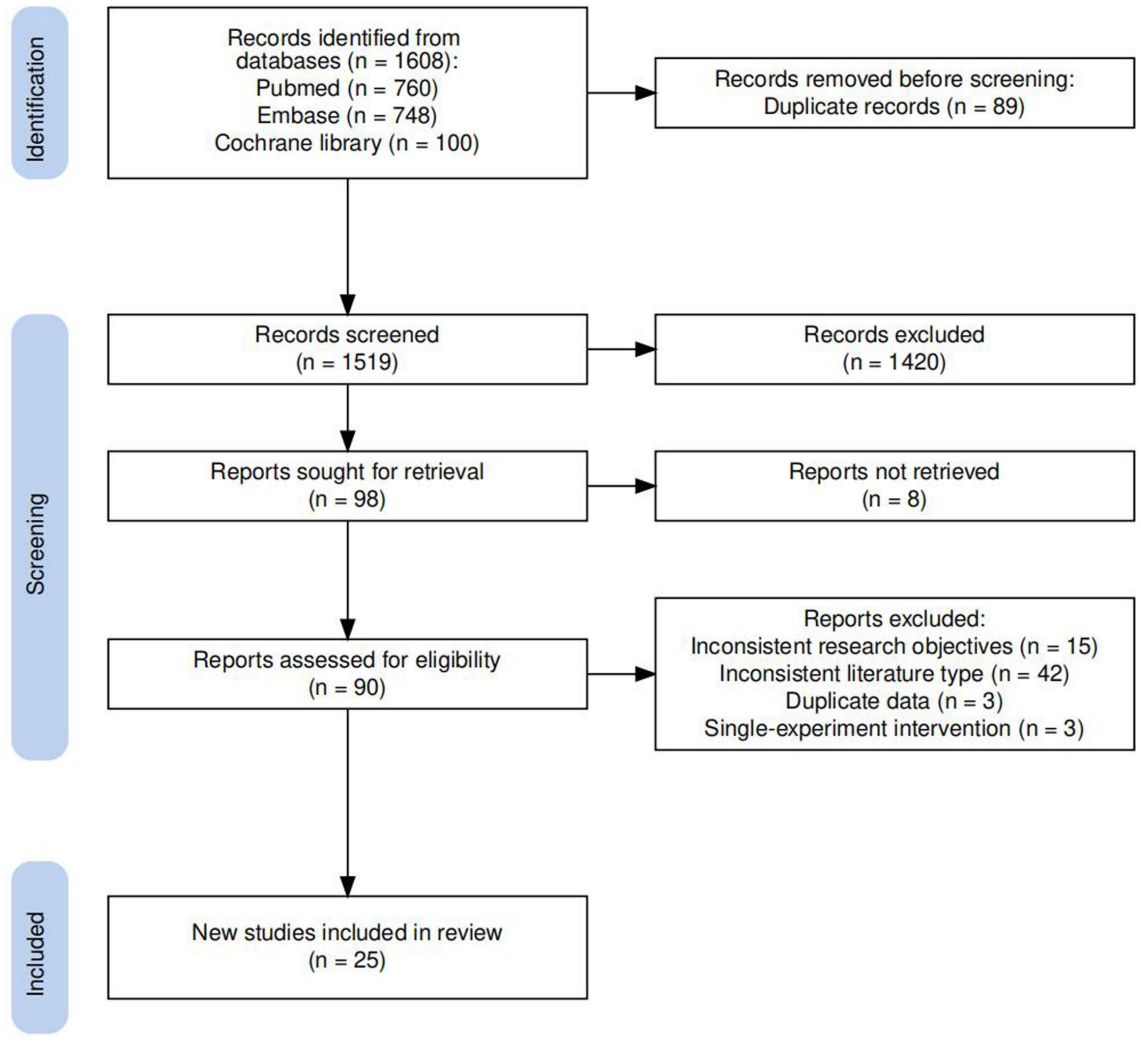
Flow diagram of screened and included studies.

**Figure 2:**
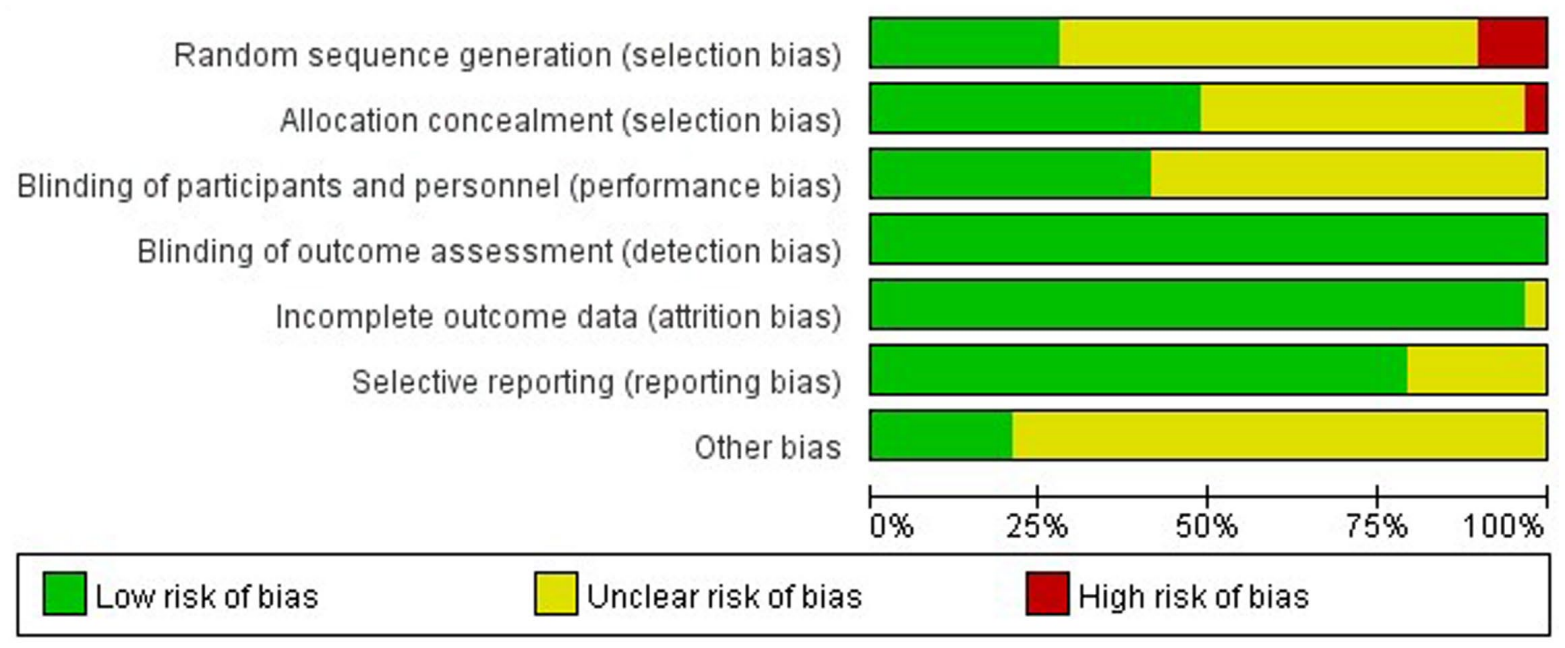
Bias assessment results of the included studies.

A total of 1,608 main articles were initially identified through data retrieval, and after removing duplicates, 1,519 articles remained. Upon careful review of titles and abstracts, 98 articles required detailed screening. Based on the inclusion and exclusion criteria, 70 publications were finally excluded from the meta-analysis. Of these, 42 studies did not conform to the RCT design, 15 studies were not relevant to the topic of our study, three studies were published by the same research team resulting in duplication of data, two studies did not provide original data and eight studies could not be accessed in full text. Finally, 25 studies were included in this meta-analysis, comprising a total of 1,256 participants. The interventions included vitamin C supplementation, vitamin E supplementation, vitamin E-coated dialyzer, ω-fatty acid supplementation, curcumin supplementation, juice supplementation, exercise intervention, and multiple antioxidant interventions. The review focused on two lipid peroxide markers: MDA and Ox-LDL. Detailed information about the included studies is presented in Table 1. The literature screening flow chart is illustrated in Figure 1, and the bias assessment of the included studies is shown in Figure 2.

**Table 1:** Basic information of the included studies.

Study	Country	Additional intervention	Intervention time	Duration of dialysis (month)	Age	Outcome indicator (source) (unit)	
						Mean ± SD	Mean ± SD
						Control	Experimental
Abdollahzad et al. (11)	Iran	Vitamin C (250 mg/day)	12 weeks	2–12	60 ± 17.10	MDA(S)(nmol/mL) 3.10 ± 1.70	MDA(S) (nmol/mL) 2.70 ± 2
Ahmadi et al. (16)	Iran	Vitamin E (400 IU/day)	2 months	16.5 ± 4.8	44.80 ± 12.70	MDA(P) (μmol/L) 6.20 ± 5.30	MDA(P) (μmol/L) 4.70 ± 1.20
Ahmadi Aet al (16).	Iran	Vitamin E (400 IU/day) + ALA (600 mg/day)	2 months	16.2 ± 5.2	53.20 ± 9.80	MDA(P) (μmol/L) 6.20 ± 5.30	MDA(P) (μmol/L) 4.50 ± 1.30
Alvarenga et al. (55)	Brazil	Curcumin (2.5 g/day)	3 months	≥3	54 ± 15	MDA (S) (μmol/L) 1.30 (0.10–2.50)	MDA (S) (μmol/L) 1.08 (0.25–4.41)
Asemi et al. (51)	Iran	ω-fatty acids (1,250 mg/day)	12 weeks	>12	18–80	MDA(P) (μmol/L) 5.70 ± 2.90	MDA(P) (μmol/L) 3.40 ± 1.80
Asemi et al. (51)	Iran	Vitamin E (400 IU/day) + ω-fatty acids (1,250 mg/day)	12 weeks	>12	54.90 ± 14.30	MDA(P) (μmol/L) 5.70 ± 2.90	MDA(P) (μmol/L) 3.40 ± 1.40
Barati Boldaji et al. (56)	Iran	Pomegranate juice (100 mL/day)	8 weeks	>3	47.80 ± 13.30	MDA (S) (μmol/L) 0.91 ± 0.01	MDA (S) (μmol/L) 0.77 ± 0.01
Candan et al. (10)	Turkey	Vitamin C (250 mg/day)	90 days	>3	45.60 (28–64)	MDA(P) (nm/g Hb) 4.16 ± 0.30	MDA(P) (nm/g Hb) 3.30 ± 1.08
Chao et al. (12)	Taiwan	Vitamin C (400 mg/day)	10 weeks	>3	57 ± 14	MDA(P) (μmol/L) 36.30 ± 24.60	MDA(P) (μmol/L) 52.90 ± 19.80
Chao et al. (12)	Taiwan	Vitamin E (400 mg/day)	10 week	>3	62 ± 8	MDA(P) (μmol/L) 36.30 ± 24.60	MDA(P) (μmol/L) 25.50 ± 19.10
Chao et al. (12)	Taiwan	Vitamin C (400 mg/day) + vitamin E (400 mg/day)	10 weeks	>3	58 ± 17	MDA(P) (μmol/L) 36.30 ± 24.60	MDA(P) (μmol/L) 32.30 ± 21.60
Daud et al. (57)	America	Vitamin E (40 mg/day)	16 weeks	>3	59 ± 12	MDA(P) (μmol/L) 4.68 ± 5.78	MDA(P) (μmol/L) 2.60 ± 2.28
Deus et al. (58)	Spain	Resistance training	6 months	54.09 ± 11.05	67.27 ± 3.24	MDA(S) (μmol/L) 14.17 ± 2.39	MDA(S) (μmol/L) 11.06 ± 2.95
Eiselt et al. (59)	Czech Republic	Vitamin C (504 mg TIW)	4 weeks	22 ± 15	61 (41–85)	MDA(P) (μmol/L) 4.28 ± 0.18	MDA(P) (μmol/L) 4.21 ± 0.16
Eiselt et al. (59)	Czech Republic	Vitamin E-coated dialyzer	4 weeks	22 ± 15	61 (41–85)	MDA(P) (μmol/L) 4.28 ± 0.18	MDA (P) (μmol/L) 3.37 ± 0.34
Eiselt et al. (59)	Czech Republic	Vitamin C (504 mg/day) + vitamin E-coated dialyzer	4 weeks	22 ± 15	61 (41–85)	MDA(P) (μmol/L) 4.28 ± 0.18	MDA (P) (μmol/L) 3.76 ± 0.13
Imani et al. (60)	France	Curcumin (1,000 mg/day)	10 weeks	46.8 ± 6	56 ± 2.50	MDA(S) (μmol/L) 4.60 ± 0.30	MDA(S) (μmol/L) 3.80 ± 0.30
Kooshki et al. (61)	France	ω-fatty acids (600 mg/day)	10 weeks	3–108	50 ± 18	MDA(S) (μmol/L) 2.60 ± 0.5	MDA(S) (μmol/L) 2.50 ± 0.50
Martins et al. (62)	Brazil	Vitamin E (250 mg/day)	8 weeks	>2	54 (53–55)	MDA(P) (μmol/L) 0.40 (0.38–0.50)	MDA(P) (μmol/L) 0.35 (0.25–0.65)
Morimoto et al. (63)	Japan	Vitamin E-coated dialyzer	6 months	≥7	69.40 ± 10.60	MDA(S) (nmol/mg LDL protein) 4.64 ± 0.96	MDA(S) (nmol/mg LDL protein) 3.16 ± 0.99
Murillo Ortiz et al. (64)	Mexico	Resveratrol + curcumin (500 mg/day)	12 weeks	57.6 ± 28.8	38.52 ± 11.14	MDA (S) (μmol/L) 70.45 ± 69.21	MDA (S) (μmol/L) 50.19 ± 32.62
Rodrigues et al. (53)	Brazil	Curcumin (1,000 mg/day)	12 weeks	50.5 (14.5–94)	48.50	MDA(P) (nmol/mL) 1.08 (0.98–1.26)	MDA(P) (nmol/mL) 0.81 (0.80–0.89)
Roozbeh et al. (65)	America	Vitamin E (400 IU/day)	3 weeks	>12	43 ± 13.40	MDA(P) (nmol/mL) 9.20 ± 2.74	MDA(P) (nmol/mL) 8.42 ± 2.63
Rusu et al. (66)	Romania	Vitamin E (600 IU/day)	8 weeks	≥3	54.80 ± 9.70	MDA(P) (nmol/mL) 2.10 ± 0.80	MDA(P) (nmol/mL) 2 ± 0.80
Sato et al. (67)	Japan	Vitamin E-coated dialyzer	Single dialysis	58.4 ± 7.2	65 ± 7.40	MDA(S) (nmol/mL) 3.58 ± 0.24	MDA(S) (nmol/mL) 3.29 ± 0.19
Shafabakhsh et al. (68)	Iran	Curcumin (80 mg/day)	12 weeks	≥3	58.30 ± 9.40	MDA(P) (μmol/L) 2.90 ± 0.90	MDA(P) (μmol/L) 2.40 ± 0.40
Shema-Didi et al. (69)	Israel	Pomegranate juice (1,000 mL TIW)	12 months	≥3	65.90 ± 11.20	MDA(S) (μmol/L) 6.90 ± 2.60	MDA(S) (μmol/L) 3.80 ± 1.30
Shimazu et al. (70)	Japan	Vitamin E-coated dialyzer	9 months	163.5 ± 91.4	59.70 ± 12.20	MDA(P) (nmol/mg LDL protein) 4.33 ± 0.96	MDA(P) (nmol/mg LDL protein) 3.46 ± 0.66
Shimazu et al. (70)	Japan	Vitamin E-coated dialyzer	9 months	163.5 ± 91.4	59.70 ± 12.20	OX-LDL (ng/μg LDL protein) 1.65 ± 0.76	Ox-LDL (ng/μg LDL protein) 1.36 ± 0.85
Sovatzidis et al. (71)	Germany	Intradialytic cardiovascular exercise	6 months	43.2 ± 13.2	52.80 ± 17.10	MDA(S) (μmol/L) 17.17 ± 4.89	MDA(S) (μmol/L) 10.52 ± 4.34
Usberti et al. (72)	Italy	Vitamin E-coated dialyzer	3 months	≥12	63 ± 11	MDA(P) (μmol/L) 1.74 ± 0.41	MDA(P) (μmol/L) 1.31 ± 0.44
Wilund et al. (73)	America	Intradialytic exercise	4 months	63.3 ± 8.7	60.80 ± 3.20	MDA(S) (μmol/L) 6.90 ± 1.31	MDA(S) (μmol/L) 5.90 ± 1.05

ALA, alpha-lipoic acid; S, serum; p, plasma; E, erythrocytes; MDA, malondialdehyde; 4-HNE, 4-hydroxynonenal; Ox-LDL: oxidized low-density lipoprotein.

### 3.2 Therapeutic efficacy of various antioxidant Intervention strategies

#### 3.2.1 The therapeutic effect of vitamin C supplementation

**Figure 3:**
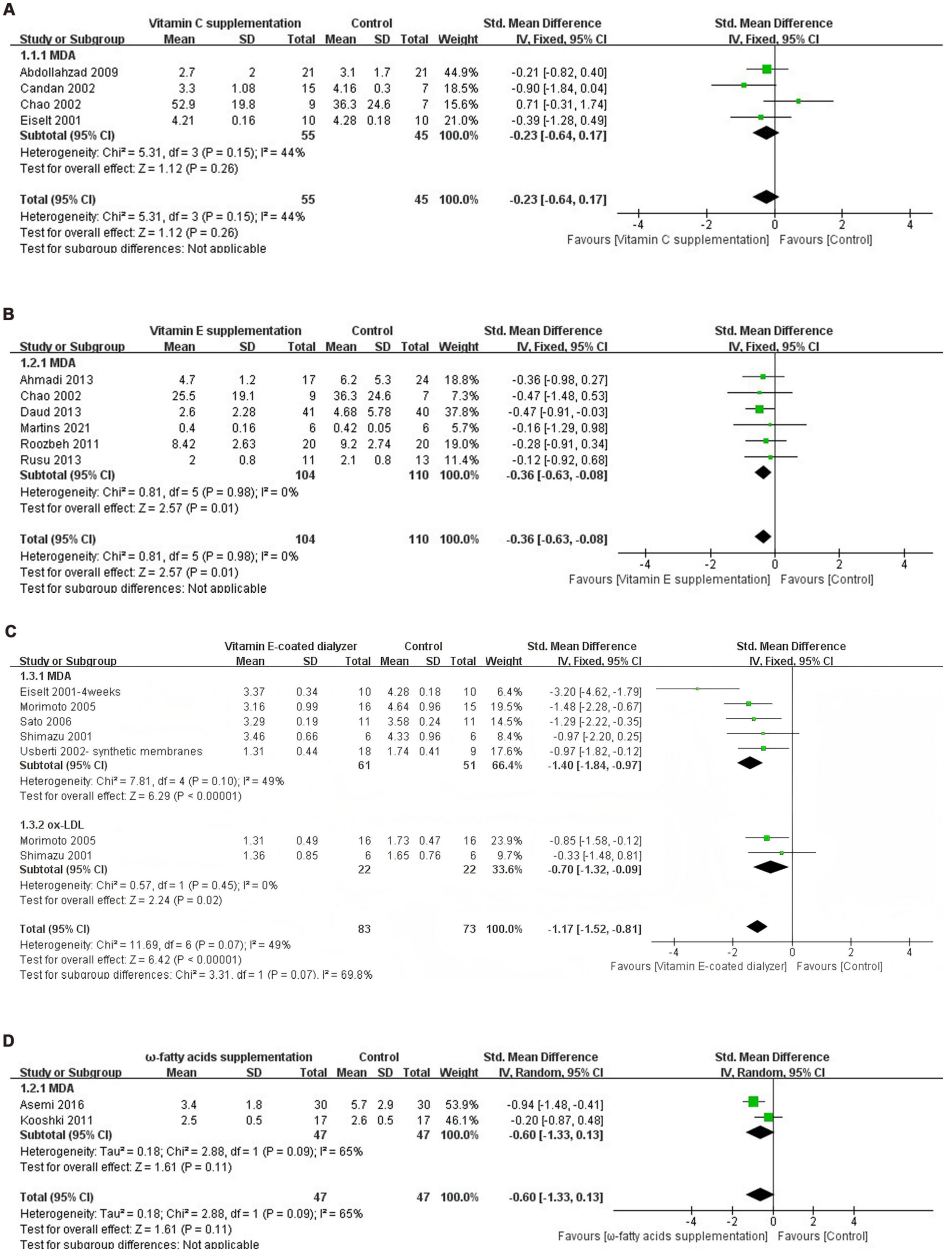
**(A)** Forest plot of vitamin C supplementation on lipid peroxide levels. **(B)** Forest plot of vitamin E supplementation on lipid peroxide levels. **(C)** Forest plot of vitamin E-coated dialyzer on lipid peroxide levels. **(D)** Forest plot of ω-fatty acids supplementation on lipid peroxide levels. **(E)** Forest plot of curcumin supplementation on lipid peroxide levels. **(F)** Forest plot of pomegranate juice supplementation on lipid peroxide levels. **(G)** Forest plot of exercise intervention on lipid peroxide levels. **(H)** Forest plot of multiple antioxidant interventions on lipid peroxide levels.

Regarding the vitamin C supplementation intervention, data from four experimental groups (*n* = 100 participants) were included in the final meta-analysis. The meta-analysis found no significant reduction in MDA levels among dialysis patients after vitamin C supplementation (SMD = −0.23, 95% CI −0.64 to 0.17, *p* = 0.26), *I*^2^ = 44%, with no significant heterogeneity observed (Figure 3A).

#### 3.2.2 The therapeutic effect of vitamin E supplementation

For the vitamin E intervention arm, the six eligible trials (*n* = 214 participants) were pooled using fixed-effects models (*I*^2^ = 0%), yielding a standardized mean difference of −0.36 (95% CI: −0.63 to −0.08, *p* = 0.01) for MDA levels reduction (Figure 3B).

#### 3.2.3 The therapeutic effect of vitamin E-coated dialyzer

Regarding the vitamin E-coated dialyzer intervention, data from six experimental groups (*n* = 156 participants) were included in the final meta-analysis. The meta-analysis showed a significant reduction in Ox-LDL levels in dialysis patients treated with a vitamin E-coated dialyzer (SMD = −0.70, 95% CI −1.32 to 0.09, *p* = 0.02). Notably, there was no heterogeneity between studies (*I*^2^ = 0%). Meanwhile, MDA levels also exhibited a significant decrease (SMD = −1.40, 95% CI −1.84 to −0.97, *p* < 0.0001), with no significant heterogeneity (*I*^2^ = 49%) (Figure 3C).

#### 3.2.4 Therapeutic effect of ω-fatty acids supplementation

Regarding the ω-fatty acids supplementation intervention, data from two experimental groups (*n* = 94 participants) were included in the final meta-analysis. The meta-analysis revealed no significant reduction in MDA levels in dialysis patients receiving ω-fatty acids supplementation (SMD = −0.60, 95% CI −1.33 to 0.13, *p* = 0.11), *I*^2^ = 65%. Given the significant heterogeneity, a random effects model was employed (Figure 3D).

#### 3.2.5 The therapeutic effect of curcumin supplementation

Regarding the curcumin supplementation intervention, data from four experimental groups (*n* = 167 participants) were included in the final meta-analysis. The meta-analysis revealed a significant reduction in MDA levels among dialysis patients following curcumin supplementation (SMD = −1.96, 95% CI −3.77 to −0.15, *p* = 0.03). However, the high *I*^2^ statistic (95%) indicated substantial heterogeneity between the included studies (Figure 3E).

#### 3.2.6 The therapeutic effect of pomegranate juice supplementation

Regarding the pomegranate juice supplementation intervention, data from two experimental groups (*n* = 142 participants) were included in the final meta-analysis. The meta-analysis did not reveal a significant decrease in MDA levels following pomegranate juice supplementation (SMD = −7.59, 95% CI −19.41 to 4.24, *p* = 0.21). Notably, there was high heterogeneity among studies (*I*^2^ = 98%) (Figure 3F).

#### 3.2.7 The therapeutic effect of exercise intervention

Regarding the pomegranate exercise therapy intervention, data from three experimental groups (*n* = 199 participants) were included in the final meta-analysis. The meta-analysis revealed a significant decrease in MDA levels following exercise therapy intervention (SMD = −1.14, 95% CI −1.44 to −0.84, *p* < 0.0001). Importantly, there was no heterogeneity between studies (*I*^2^ = 0%), allowing for the use of a fixed-effects model (Figure 3G).

#### 3.2.8 The therapeutic effect of multiple antioxidant interventions

Regarding the pomegranate multiple antioxidant interventions, data from five experimental groups (*n* = 184 participants) were included in the final meta-analysis. The meta-analysis results demonstrated a significant decrease in MDA levels among dialysis patients following multiple antioxidant interventions (SMD = −0.85, 95% CI −1.15 to −0.19, *p* = 0.01), *I*^2^ = 75%, with heterogeneity (Figure 3H).

### 3.3 Subgroup analysis

The results of the meta-analysis indicated high heterogeneity in interventions involving curcumin supplementation and multiple antioxidant interventions. To identify the source of this heterogeneity, MDA levels were stratified by study source, sample source, sample size, intervention time, male proportion, age, dialysis duration and whether diabetes is excluded.

In addition, we performed the subgroup analyses for various antioxidant supplementation doses to investigate the possible effects of supplementation dose.

#### 3.3.1 Subgroup analysis of high heterogeneity

Subgroup analysis of MDA levels after curcumin supplementation found no significant change in heterogeneity and *p*-value, demonstrating that the obtained results were stable (Table 2).

**Table 2:** Results of subgroup analysis of curcumin supplementation on MDA levels.

Subgroup		*n* (cases)	Sample size	SMD (95% CI)	Value	Heterogeneity	
						*I*^2^ (%)	*p*-value
Research source	America	2	68	−1.62 (−3.48 to 0.24)	12.51	92	<0.0001
	Asian	2	88	−2.37 (−7.50 to 2.75)	48.90	98	<0.0001
Sample source	Plasma	3	120	−1.76 (−4.02 to 0.50)	49.90	96	<0.0001
	Serum	1	36	−2.61 (−3.52 to −1.69)	0		
Sample size	≥20	2	92	−2.82 (−7.04 to 1.40)	38.11	97	<0.0001
	<20	2	64	−1.18 (−3.95 to 1.58)	22.07	96	<0.0001
Intervention time	≥3 months	3	120	−1.76 (−4.02 to 0.50)	49.90	96	<0.0001
	>3 months	1	36	−1.96 (−3.77 to −1.69)	0		
Male proportion	>50%	3	128	−2.72 (−5.05 to −0.38)	43.9	95	<0.0001
	50%	1	28	−1.96 (−3.77 to −0.15)	0		
Dialysis duration	≥3 years	2	76	−3.77 (−6.13 to −1.41)	9.16	89	0.002
	<3 years	2	80	−0.29 (−1.19 to 0.62)	3.97	75	0.046

Subgroup analysis stratified by study source indicated no significant decline in MDA levels among dialysis patients in Asia (SMD = −0.62, 95% CI −1.09 to −0.15, *p* = 0.22) (SMD = −0.57, 95% CI −0.90 to −0.24, *p* = 0.35), with an inter-study heterogeneity of 35%. Meanwhile, it indicated significant decline in MDA levels among dialysis patients in America (SMD = −1.7, 95% CI −1.51 to −0.19, *p* < 0.0001), with an inter-study heterogeneity of 0%. This suggests that race might contributing to the observed heterogeneity. Sample size stratified subgroup analysis indicated that MDA levels in dialysis patients with a sample size ≥40 (SMD = −0.62, 95% CI −1.02 to −0.22, *P* = 0.23) did not show a significant reduction. Heterogeneity remained moderate (*I*^2^ = 31%). Conversely, patients in studies with a sample size less than 40 exhibited a significant decline in MDA levels following intervention (SMD = −0.93, 95% CI −1.74 to −0.13, *p* = 0.003). Furthermore, MDA levels in dialysis patients significantly reduced when diabetes was uncontrolled (SMD = −1.62, 95% CI −4.57 to −1.33, *p* = 0.001). In contrast, there was no significant reduction in MDA levels in dialysis patients with controlled diabetes (SMD = −0.62, 95% CI −1.02 to −0.22, *p* = 0.23), and the heterogeneity among the studies including controlled diabetes was 31%. Subgroup analyses revealed that patients with extended dialysis duration (>3 years) demonstrated markedly greater MDA level reductions (SMD = −1.70, 95% CI −4.44 to 1.05) compared to those with shorter dialysis duration (SMD = −0.62, 95% CI −1.09 to −0.15) (Table 3).

**Table 3:** Results of subgroup analysis of multiple antioxidant interventions on MDA levels.

Subgroup		*n* (cases)	Sample size	SMD (95% CI)	Value	Heterogeneity	
						*I*^2^ (%)	*p*-value
Research source	Asian	3	188	−0.62 (−1.09 to −0.15)	3.08	35	0.22
	America	2	80	−1.70 (−1.51 to −0.19)	12.79	0	<0.0001
Sample source	Plasma	4	228	−1.03 (−1.89 to −0.16)	14.64	79.5	0.002
	Serum	1	40	−0.37 (−0.99 to 0.26)	0		
Sample size	≥40	3	148	−0.62 (−1.02 to −0.22)	2.91	31	0.23
	<40	2	120	−1.62 (−1.51 to −0.19)	18.0	72	0.003
Intervention time	≥2 months	2	80	−0.70 (−1.32 to −0.09)	2.24	55.3	0.13
	<2 months	3	188	−3.17 (−4.57 to −1.77)	13.86	86	0.001
Diabetes	Control	2	120	−0.62 (−1.02 to −0.22)	2.91	31	0.23
	Uncontrolled	3	148	−1.62 (−4.57 to 1.33)	11.79	92	0.001
Age	≥60 years	1	40	−3.17 (−4.58 to −1.77)	0		
	<60 years	4	228	−0.57 (−0.92 to −0.21)	3.68	18	0.298
Dialysis duration	≥3 years	2	80	−1.70 (−4.44 to 1.05)	12.8	92.2	<0.001
	<3 years	3	188	−0.62 (−1.09 to −0.15)	3.1	35	0.347

#### 3.3.2 Meta-regression analysis of supplemental dose

Meta-regression analyses were conducted to evaluate potential dose–response relationships between antioxidant supplementation (vitamin C, vitamin E, and curcumin) and observed heterogeneity. Pre-specified covariates included daily dosage (mg/day), with all models adjusted for baseline oxidative status. The results revealed no significant dose-response relationships between antioxidant supplementation (vitamin C: *p* = 0.68, 95% CI −0.0130 to 0.0163; vitamin E: *p* = 0.56, 95% CI −0.0021 to 0.0034; curcumin: *p* = 0.692, 95% CI −0.0059 to 0.0073).

### 3.4 Sensitivity analysis

**Figure 4:**
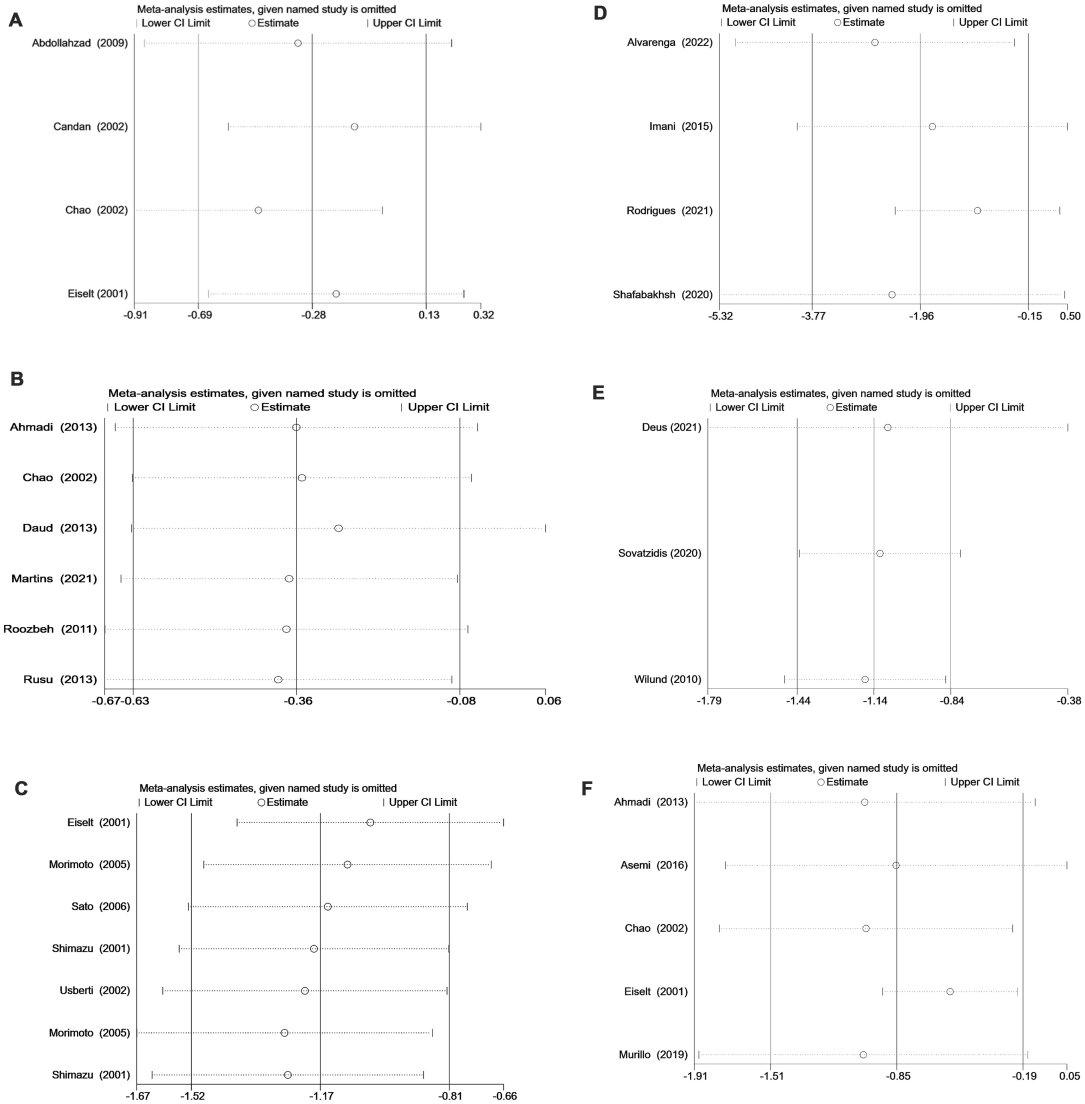
**(A)** Sensitivity analysis of vitamin C supplementation. **(B)** Sensitivity analysis of vitamin E supplementation. **(C)** Sensitivity analysis of vitamin E-coated dialyzer therapy. **(D)** Sensitivity analysis of curcumin supplementation. **(E)** Sensitivity analysis of exercise intervention. **(F)** Sensitivity analysis of multiple antioxidant interventions.

To assess the robustness of the meta-analysis results, sensitivity analyses were performed for studies that included more than two articles. The outcomes revealed that no individual studies significantly influenced the combined results, indicating the statistical robustness of the findings (Figure 4).

### 3.5 Publication bias

To assess publication bias, we incorporated funnel plot analyses, which revealed no significant asymmetry. Given the limited number of studies (*n* < 10) and the associated low statistical power for detecting asymmetry, we complemented the funnel plot analysis with Egger’s regression test (Table 4). The combined results from both methods consistently indicated the absence of significant publication bias. While our comprehensive bias assessment framework suggested minimal publication bias, the limited statistical power inherent in small meta-analyses necessitates cautious interpretation. Future updates with additional trials are warranted to confirm these observations.

**Table 4:** Egger test for each intervention.

Additional intervention	*t*	*p* > |*t*|	95% CI
Vitamin C	0.11	0.922	−16.404 to 17.271
Vitamin E	−0.91	0.398	−4.406 to 2.017
Vitamin E-coated dialyzer	−1.00	0.362	−8.822 to 3.872
Curcumin	−1.87	0.159	−26.458 to 6.903
Pomegranate juice	−2.59	0.234	−38.133 to 25.199
Exercise intervention	0.8	0.473	−5.912 to 8.940
Multiple interventions	−1.76	0.153	−9.293 to 2.085

## 4 Discussion

In this study, we conducted a comprehensive meta-analysis of existing RCT studies focusing on antioxidant intervention in dialysis patients. Our findings highlight several significant conclusions: supplementation with vitamin E, vitamin E-coated dialyzer treatment, curcumin supplementation, exercise intervention, and multiple antioxidant interventions were all found to effectively improve the lipid peroxidation status of dialysis patients. However, Supplementation with vitamin C, supplementation with ω-fatty acids and pomegranate juice did not significantly improve the lipid peroxidation status of dialysis patients.

Vitamin C and vitamin E are key antioxidants known for their role in protecting LDL cholesterol from free radical damage (29, 30). However, the use of vitamin C in clinical practice is currently controversial. Our results showed that vitamin C supplementation did not significantly reduce MDA levels in dialysis patients. Meanwhile, our findings indicate that the dose of vitamin C did not significantly impact the outcomes of this study. Interestingly, one study indicated a potential increase in cardiovascular mortality with high-dose vitamin C supplementation (≥300 mg/day) (31). In addition, Chao et al. (12), found that plasma lipid peroxidation levels were significantly reduced in the vitamin C supplement group at week 6. Further studies are needed to generate cumulative time-dependent and dose- dependent data to confirm the effect of vitamin C on lipid peroxidation. In addition, micronutrients can induce synergistic effects. For instance, vitamin C enhances the lipid antioxidant effects of vitamin E (32). Chao et al. (12) uncovered that vitamin C and vitamin E supplementation significantly reduced lipid peroxidation levels in dialysis patients. As such, vitamin E may exert synergistic antioxidant effects on vitamin C. In addition, this study demonstrates that vitamin E supplementation can improve lipid peroxidation damage in dialysis patients. Lipid peroxidation occurs in three distinct stages: initiation, propagation, and termination (20). Vitamin E acts as a chain-breaking antioxidant by scavenging alkylperoxyl radicals, interrupting the chain reaction and preventing further damage (33). Moreover, it disrupts the reproductive step by forming vitamin E free radicals, scavenging lipid peroxy free radicals, which are further scavenged by other antioxidants (34). Currently, there is a meta-analysis investigating the therapeutic effect of vitamin E supplementation on OS in hemodialysis patients (15). However, not all the studies included in that meta-analysis met the rigorous criteria of RCTs, leading to high heterogeneity and subsequently, controversy regarding the validity of the results. In our study, we carefully curated relevant RCT studies, ensuring a higher standard of quality. We found that vitamin E supplementation significantly reduced in MDA levels among dialysis patients, with no significant heterogeneity. Although a review of the literature suggests that oral vitamin E is generally well-tolerated, the safety of high-dose vitamin E remains a subject of ongoing debate (35). In the subgroup analysis of doses, we found that low dose vitamin E supplementation significantly improved lipid peroxidation while higher doses of vitamin E did not. The effective dose range of clinical vitamin E supplementation awaits the results of long-term, large controlled clinical trials.

During HD, blood contact with bioincompatible dialyzer membranes and dialysate triggers the activation of complement factors, platelets, and polymorphonuclear cells, leading to the production of ROS and exacerbating OS (36, 37). Vitamin E might inhibit platelet activation, thereby limiting platelet adhesion (38, 39). Clermont et al. (40) found a significantly lower rate of neutrophil activation induced by novel vitamin E-coated dialyzer compared to highly biocompatible synthetic dialysis membranes. This suggests that vitamin E coating on dialyzer may reduce OS exacerbation by inhibiting neutrophil and platelet activation. This study revealed that vitamin E-coated dialyzer effectively reduced Ox-LDL levels in dialysis patients, aligning with the findings of Yang et al. (41). Our findings regarding the effectiveness of vitamin E-coated dialyzers in reducing MDA levels in dialysis patients align with the conclusions of Sosa et al. (42) and D’Arrigo et al. (43). However, we note that their studies may have included a wider range of research designs, not just RCTs. In contrast, our meta-analysis exclusively focused on RCTs, which are considered the gold standard for establishing causal relationships. This methodological rigor strengthens the credibility of our conclusion: vitamin E-coated dialyzers likely offer a significant benefit in reducing lipid peroxidation in dialysis patients. Although vitamin E-coated dialyzers have shown potential benefits for dialysis patients, their broad clinical adoption may be limited by their higher cost compared to uncoated dialyzers. To fully realize the potential benefits of this technology, collaborative efforts between biomaterial scientists, entrepreneurs, and nephrologists are essential.

Curcumin is a potent inhibitor of ROS production by providing hydrogen, thereby reducing lipid peroxidation damage (44). In addition, curcumin can activate Nrf2, a key antioxidant stress protein (45). Curcumin has been shown to improve outcomes in numerous diseases, such as autoimmune diseases (46), diabetes mellitus (47), and fatty liver diseases (48). This study reveals that targeting anti-lipid peroxidation may improve the outcomes of dialysis patients. In addition, we uncovered that high doses of curcumin have stronger anti-lipid peroxidation effects. Curcumin is a nontoxic, non-mutagenic, non-carcinogenic, non-photo toxic agent. Studied investigating the safety of turmeric, showed that standardized powder of turmeric and curcumin are safe for human use. However, whether the antioxidant effect of curcumin is dose-dependent needs to be investigated in larger studies.

Furthermore, this study also found that exercise intervention significantly declined MDA levels in dialysis patients. This exercise-mediated effect can be explained by the excitation theory, which posits that regular exercise leads to intermittent and transient ROS production and OS, stimulating redox-sensitive signaling pathways. This stimulation promotes protective adaptation, preparing the body to cope with subsequent higher ROS levels and molecular damage (49). However, acute exercise induces more OS (50). Therefore, under the premise of safety, dialysis patients should be encouraged to engage in long-term, low-to-moderate-intensity exercise training whenever possible. Clinicians should be encouraged to develop personalized exercise programs tailored to the specific needs of their patients.

The multiple antioxidant interventions in this study involved the simultaneous application of two antioxidant measures. In four out of the five studies, vitamin E was co-supplemented with another antioxidant. The study of Asemi et al. (51) revealed that supplementation of ω-fatty acids alone did not improve the lipid peroxidation status of dialysis patients. However, when combined with vitamin E, ω-fatty acids significantly reduced lipid peroxidation levels in patients. Given that ω-fatty acids are sensitive to oxidation, they may exhibit more efficacy when used with vitamin E rather than independently. Chao et al. (12) demonstrated that the combination of vitamin C and vitamin E effectively improved the antioxidant status of dialysis patients, with the effects persisting for an extended period even after supplement termination. Consequently, in cases where single antioxidant intervention proves ineffective, exploring combinations of multiple antioxidants could be considered to enhance the lipid peroxidation status of dialysis patients.

Multidimensional subgroup analyses were performed in the curcumin and multi-antioxidant intervention groups, both exhibiting substantial heterogeneity (*I*^2^ > 50%). Notwithstanding the absence of significant moderators in subgroup analyses of curcumin, the stratified evaluation of MDA levels revealed persistent homogeneity following curcumin intervention. At the same time, we found race might contributing to the observed heterogeneity. This methodological triangulation confirmed the stability of anti-peroxidation effects across population subsets.

Sensitivity analysis indicated minimal fluctuations in heterogeneity and results across the included studies for each intervention, suggesting that the conclusions of this study were statistically robust. In addition, for each intervention, the Egger test revealed that there was no publication bias, which indicated strong robustness of the conclusions of our study. However, there are several limitations in this study. Bias risk assessment identified some potential selection bias. However, according to the Cochrane Handbook, a high risk of bias does not necessarily equate to low-quality evidence. Subsequent sensitivity analyses, excluding studies with high-bias risk, did not alter our conclusions. The RCTs included in the study had small sample sizes, including only 1,256 subjects across 25 articles. Consequently, larger clinical trials are imperative to validate these findings. Furthermore, most assays to determine MDA have been developed on the basis of its derivatization with thiobarbituric acid (TBA), which is insufficiently sensitive and disturbed by too much interference coming from MDA related species or overestimation derived from stressing analysis conditions. Although there has been significant improvement of methods for MDA detection, they have been fully validated (52). Unfortunately, among the studies included in this study, only Rodrigues et al. (53) used the detection method that utilizes non-thiobarbituric acid (TBA) derivatization to overcome the biases associated with derivatization of MDA with TBA. Meanwhile, due to the backwardness of MDA determination methods in the literatures included in this review, all of which indicated that blood samples taken were centrifuged and stored until assay. This leads to limitations in the results of our study. A recent study has shown that plasmonic optical fiber biosensor for point-of-care detection of MDA has been developed (54), and the application of this technology is expected to solve this important issue. Given its importance as a marker of lipid peroxidation and its potential harmful effects on health, the use of new and reliable assays to measure MDA should be encouraged in future medical trials.

## 5 Conclusion

Supplementation of vitamin E, vitamin E-coated dialyzer treatment, curcumin supplementation, exercise intervention, and multiple antioxidant interventions can effectively reduce the level of lipid peroxidation biomarkers in dialysis patients. However, due to the limitations of the assays included in our study, it is hoped that designers of future clinical trials will take this into account and actively apply improved assays.

## Data Availability

The original contributions presented in the study are included in the article/Supplementary material, further inquiries can be directed to the corresponding author.
