# Supplementary figures and images for: Efficacy of antioxidant intervention and exercise intervention for lipid peroxidation in dialysis patients: a meta-analysis
Source: Front Med (Lausanne). 2025 Mar 17;12:1473818. doi: 10.3389/fmed.2025.1473818 (PMC11955636; doi:10.3389/fmed.2025.1473818)

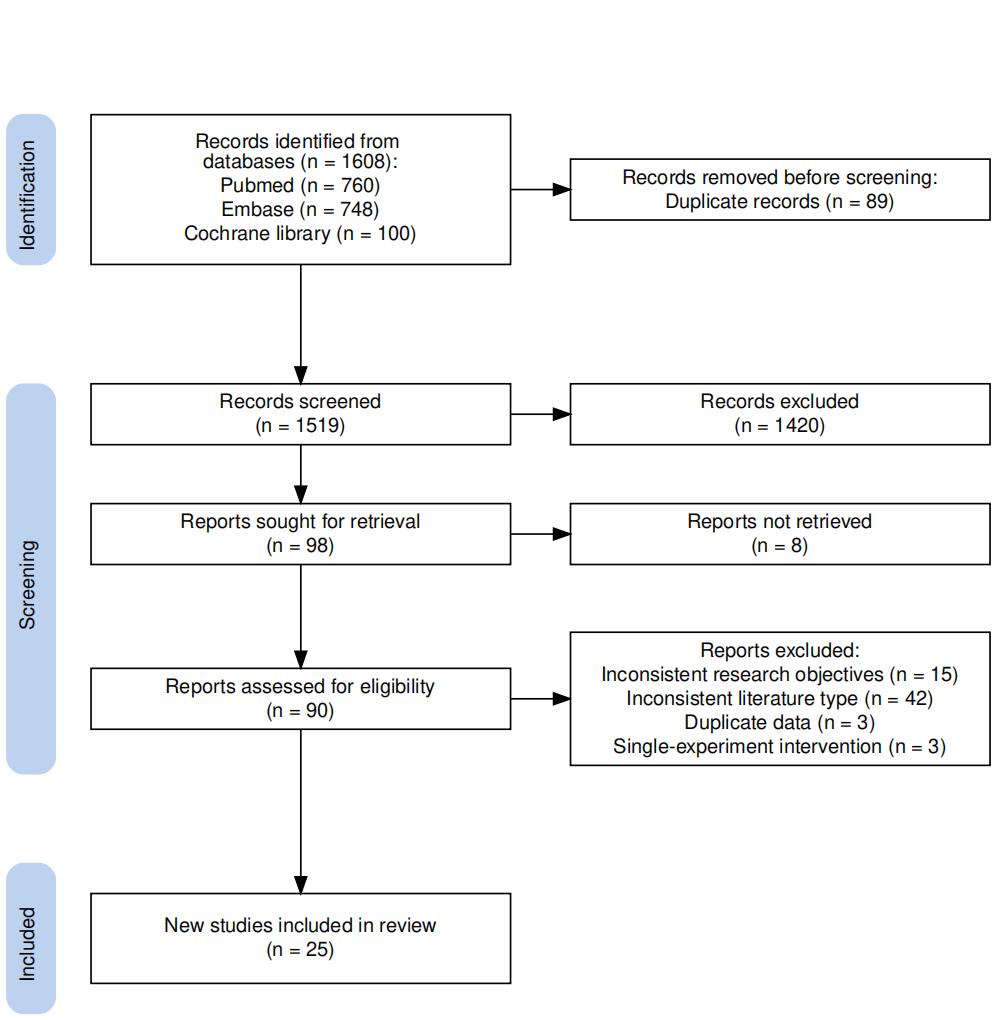

Supplement: Supplementary file 1 [file Data_Sheet_1.zip › Supplementary Material Presentation/Supplementary_Figures/Fig. 1 Flow diagram of screened and included studies.jpg]

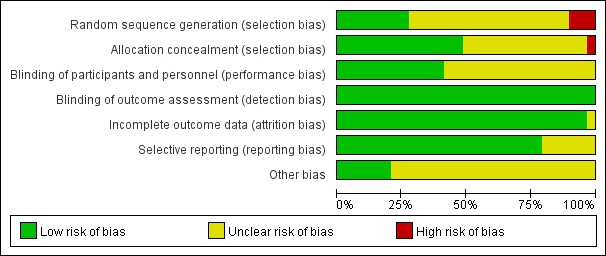

Supplement: Supplementary file 1 [file Data_Sheet_1.zip › Supplementary Material Presentation/Supplementary_Figures/Fig. 2 Bias assessment results of the included studies .jpg]

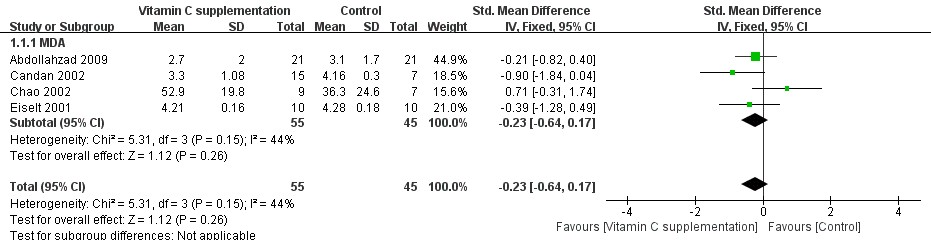

Supplement: Supplementary file 1 [file Data_Sheet_1.zip › Supplementary Material Presentation/Supplementary_Figures/Fig. 3a Forest plot of vitamin C supplementation on lipid peroxide levels.jpg]

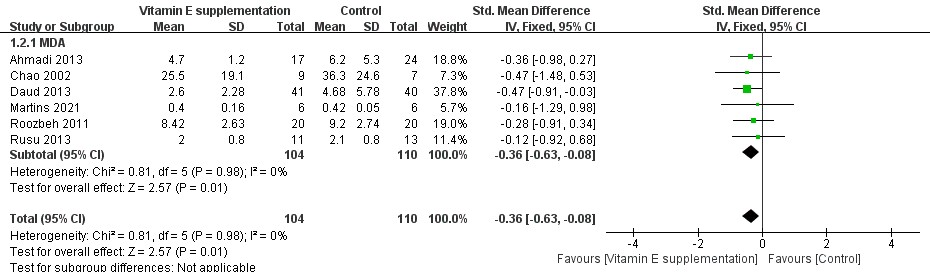

Supplement: Supplementary file 1 [file Data_Sheet_1.zip › Supplementary Material Presentation/Supplementary_Figures/Fig. 3b Forest plot of vitamin E supplementation on lipid peroxide levels.jpg]

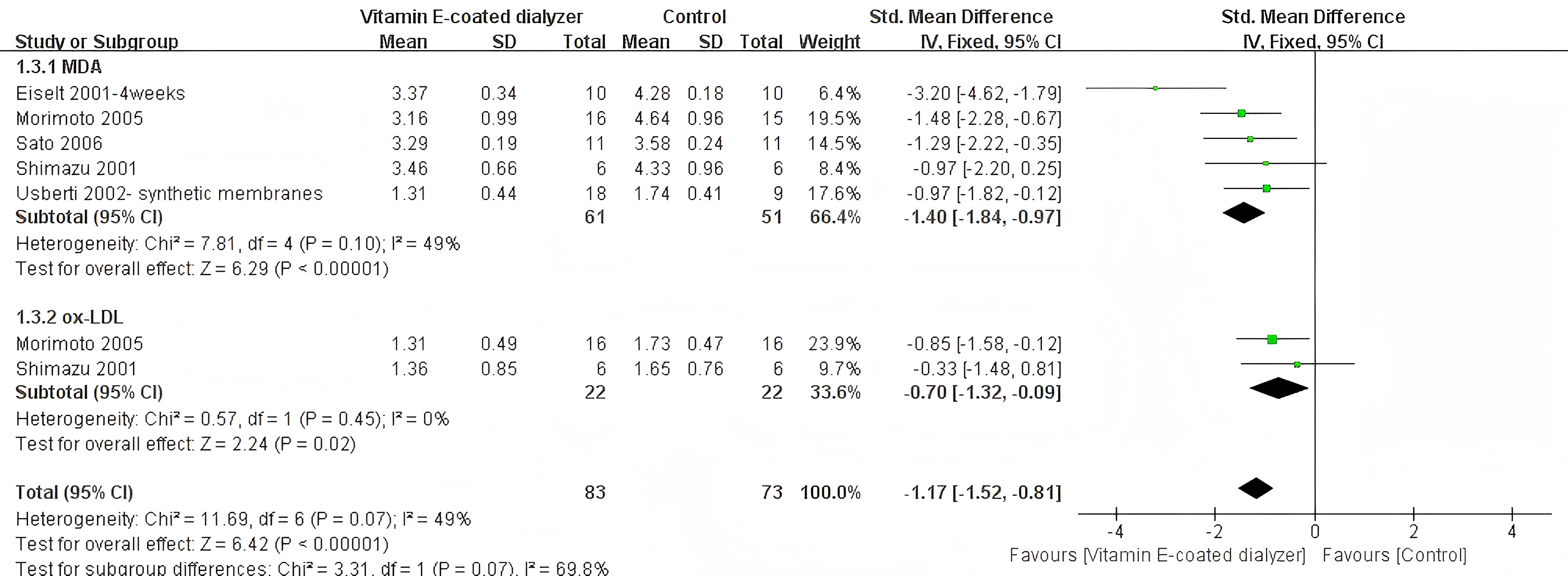

Supplement: Supplementary file 1 [file Data_Sheet_1.zip › Supplementary Material Presentation/Supplementary_Figures/Fig. 3c Forest plot of vitamin E-coated dialyzer on lipid peroxide levels.jpg]

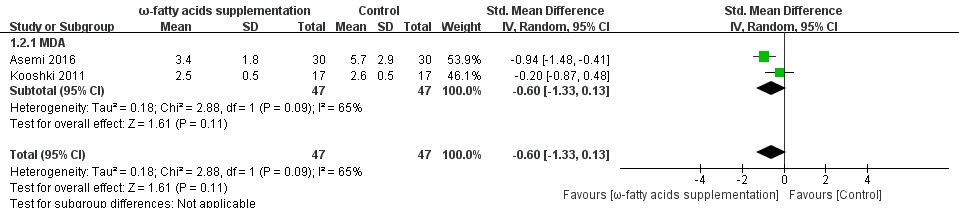

Supplement: Supplementary file 1 [file Data_Sheet_1.zip › Supplementary Material Presentation/Supplementary_Figures/Fig. 3d Forest plot of ω-fatty acids supplementation on lipid peroxide levels.jpg]

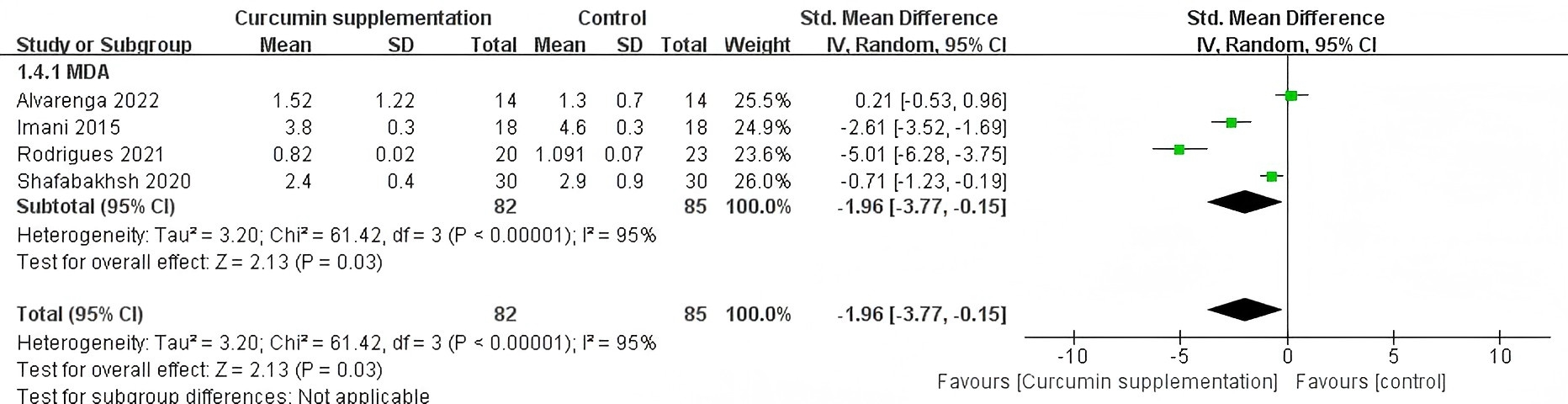

Supplement: Supplementary file 1 [file Data_Sheet_1.zip › Supplementary Material Presentation/Supplementary_Figures/Fig. 3e Forest plot of curcumin supplementation on lipid peroxide levels.jpg]

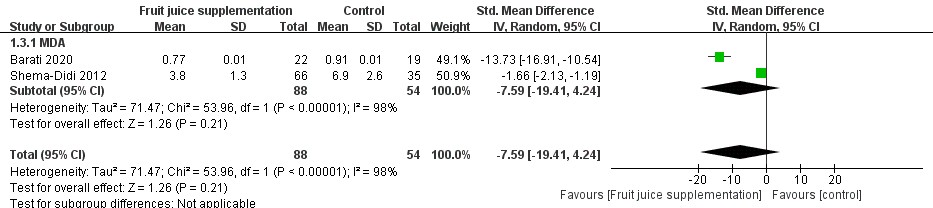

Supplement: Supplementary file 1 [file Data_Sheet_1.zip › Supplementary Material Presentation/Supplementary_Figures/Fig. 3f Forest plot of pomegranate juice supplementation on lipid peroxide levels.jpg]

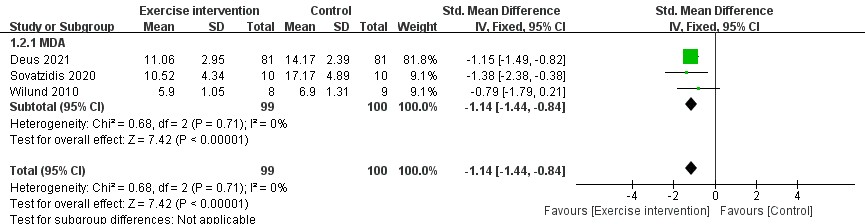

Supplement: Supplementary file 1 [file Data_Sheet_1.zip › Supplementary Material Presentation/Supplementary_Figures/Fig. 3g Forest plot of exercise intervention on lipid peroxide levels.jpg]

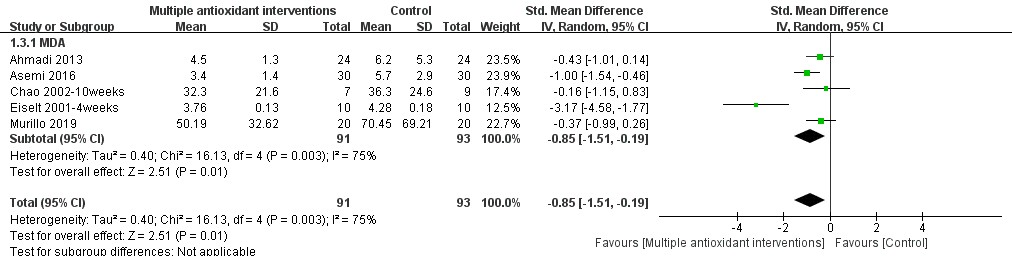

Supplement: Supplementary file 1 [file Data_Sheet_1.zip › Supplementary Material Presentation/Supplementary_Figures/Fig. 3h Forest plot of multiple antioxidant interventions on lipid peroxide levels.jpg]

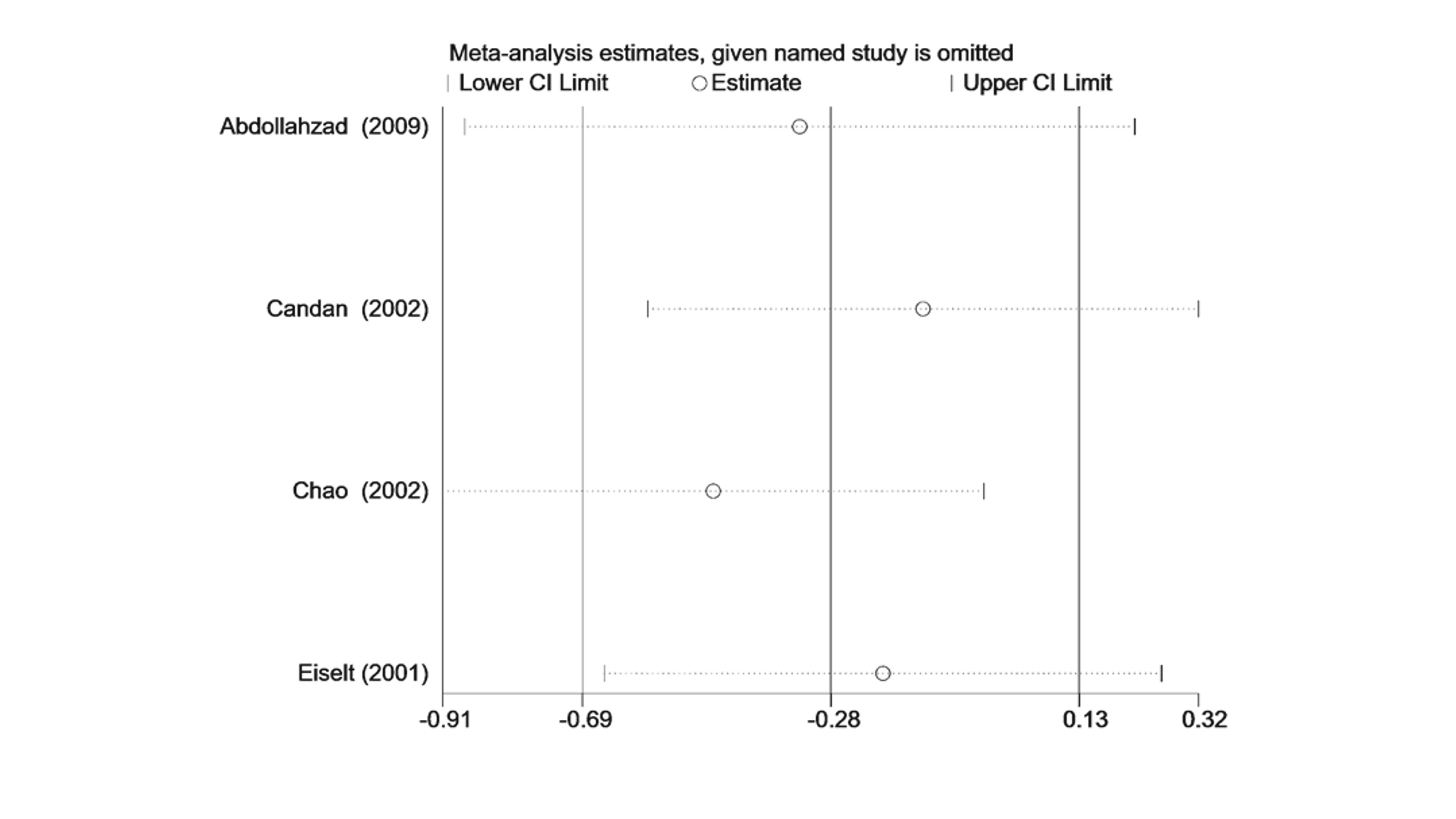

Supplement: Supplementary file 1 [file Data_Sheet_1.zip › Supplementary Material Presentation/Supplementary_Figures/Fig. 4a Sensitivity analysis of vitamin C supplementation.jpg]

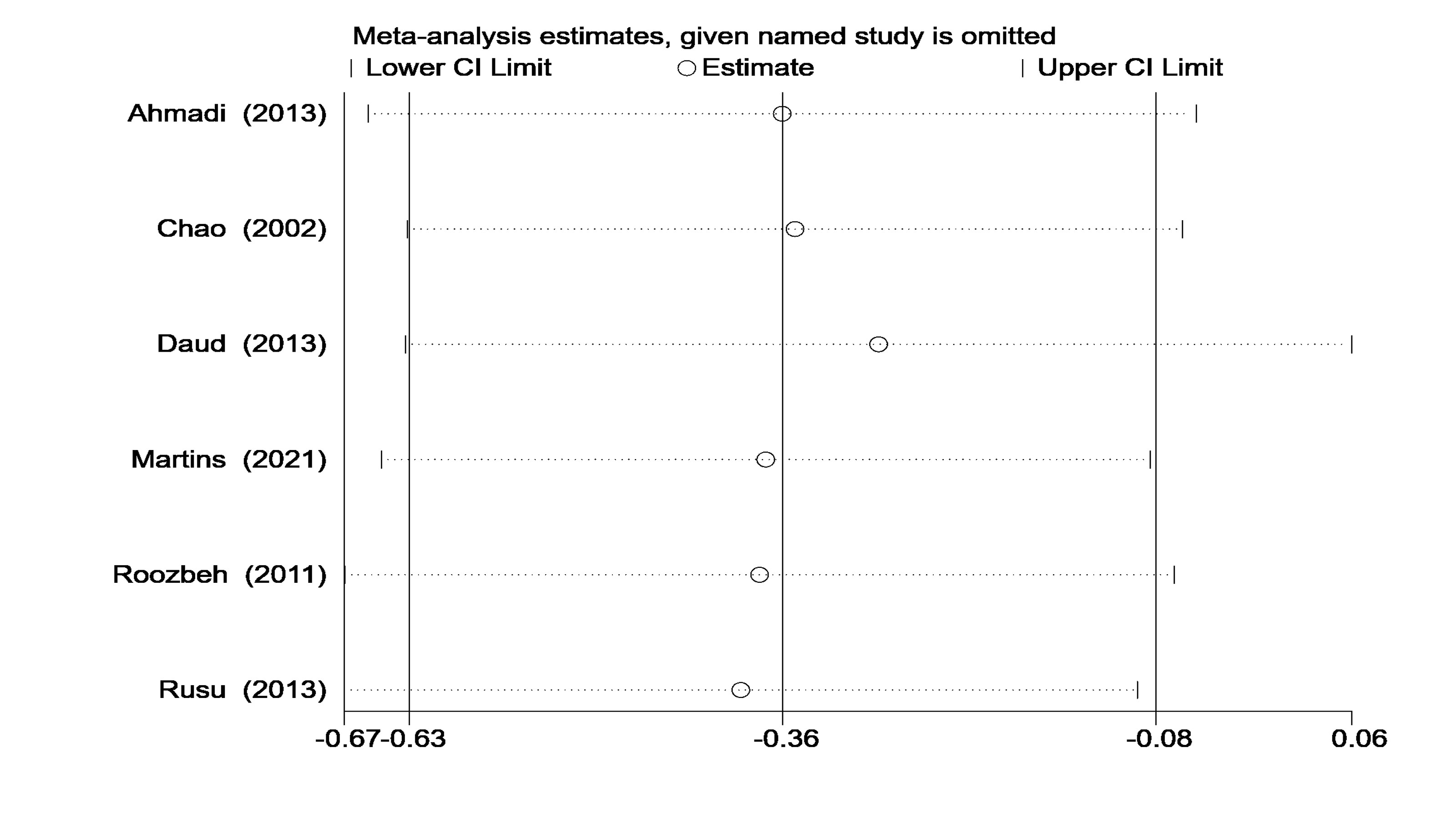

Supplement: Supplementary file 1 [file Data_Sheet_1.zip › Supplementary Material Presentation/Supplementary_Figures/Fig. 4b Sensitivity analysis of vitamin E supplementation.jpg]

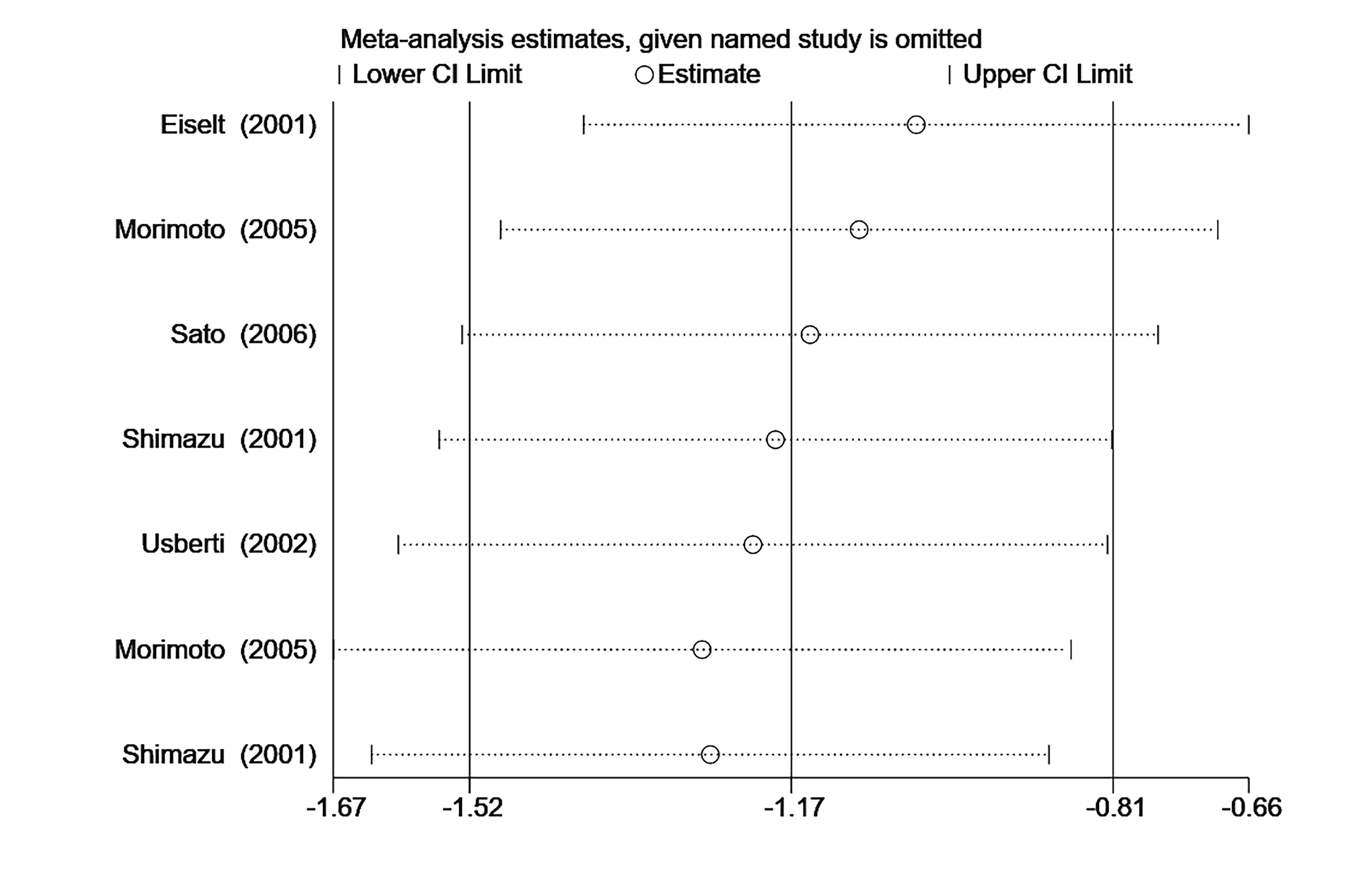

Supplement: Supplementary file 1 [file Data_Sheet_1.zip › Supplementary Material Presentation/Supplementary_Figures/Fig. 4c Sensitivity analysis of vitamin E-coated dialyzer therapy.jpg]

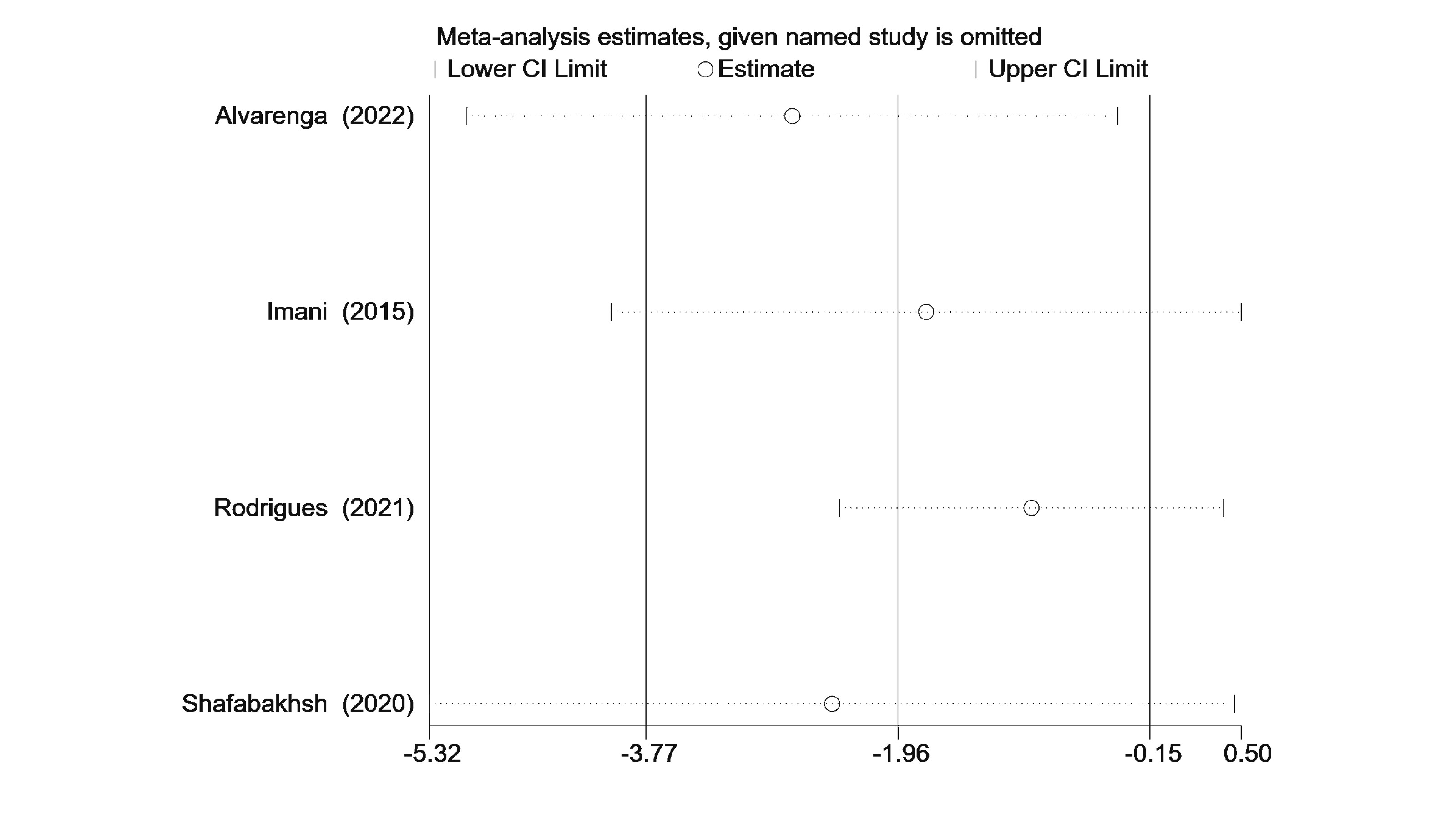

Supplement: Supplementary file 1 [file Data_Sheet_1.zip › Supplementary Material Presentation/Supplementary_Figures/Fig. 4d Sensitivity analysis of curcumin supplementation.jpg]

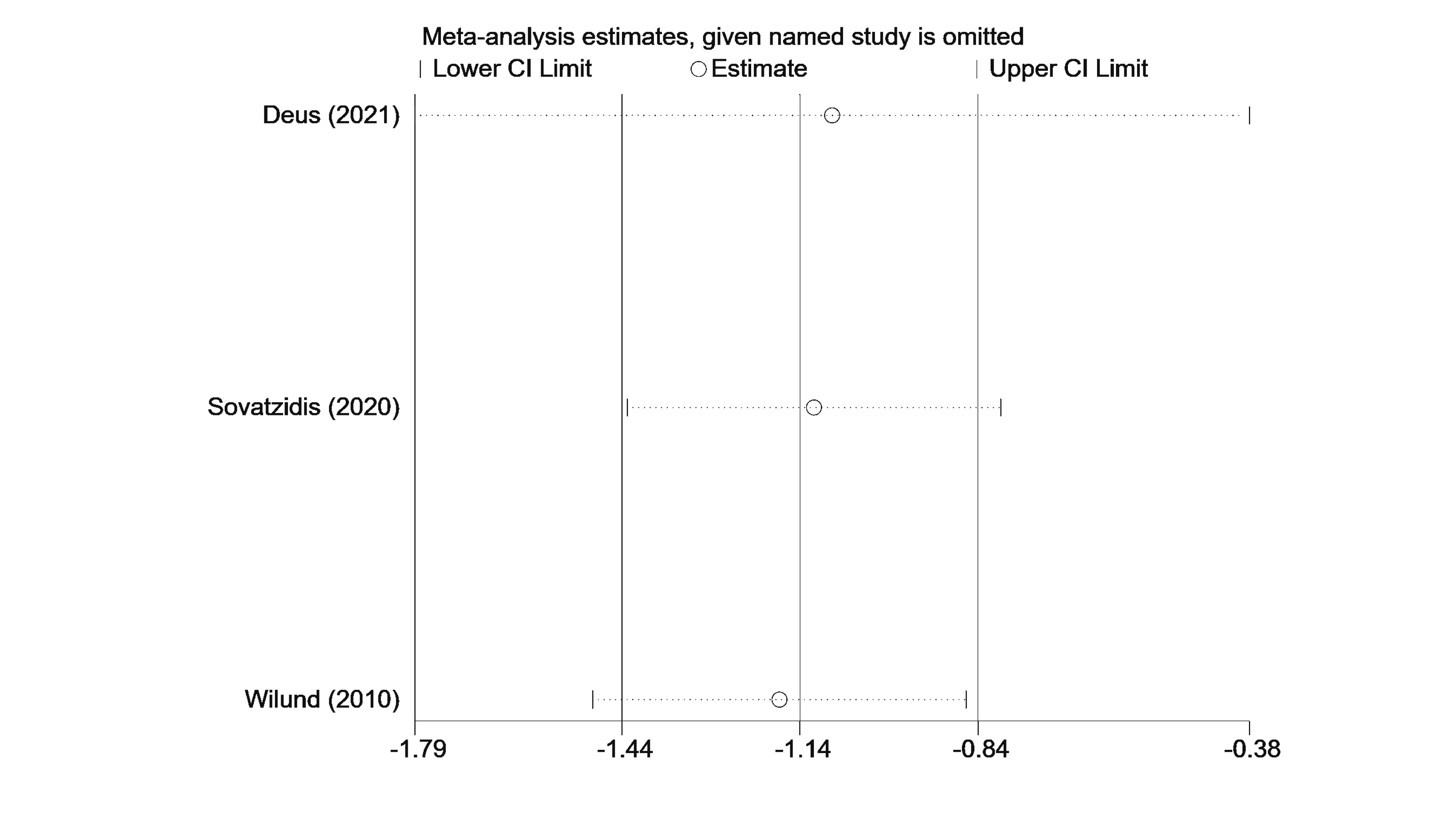

Supplement: Supplementary file 1 [file Data_Sheet_1.zip › Supplementary Material Presentation/Supplementary_Figures/Fig. 4e Sensitivity analysis of exercise intervention.jpg]

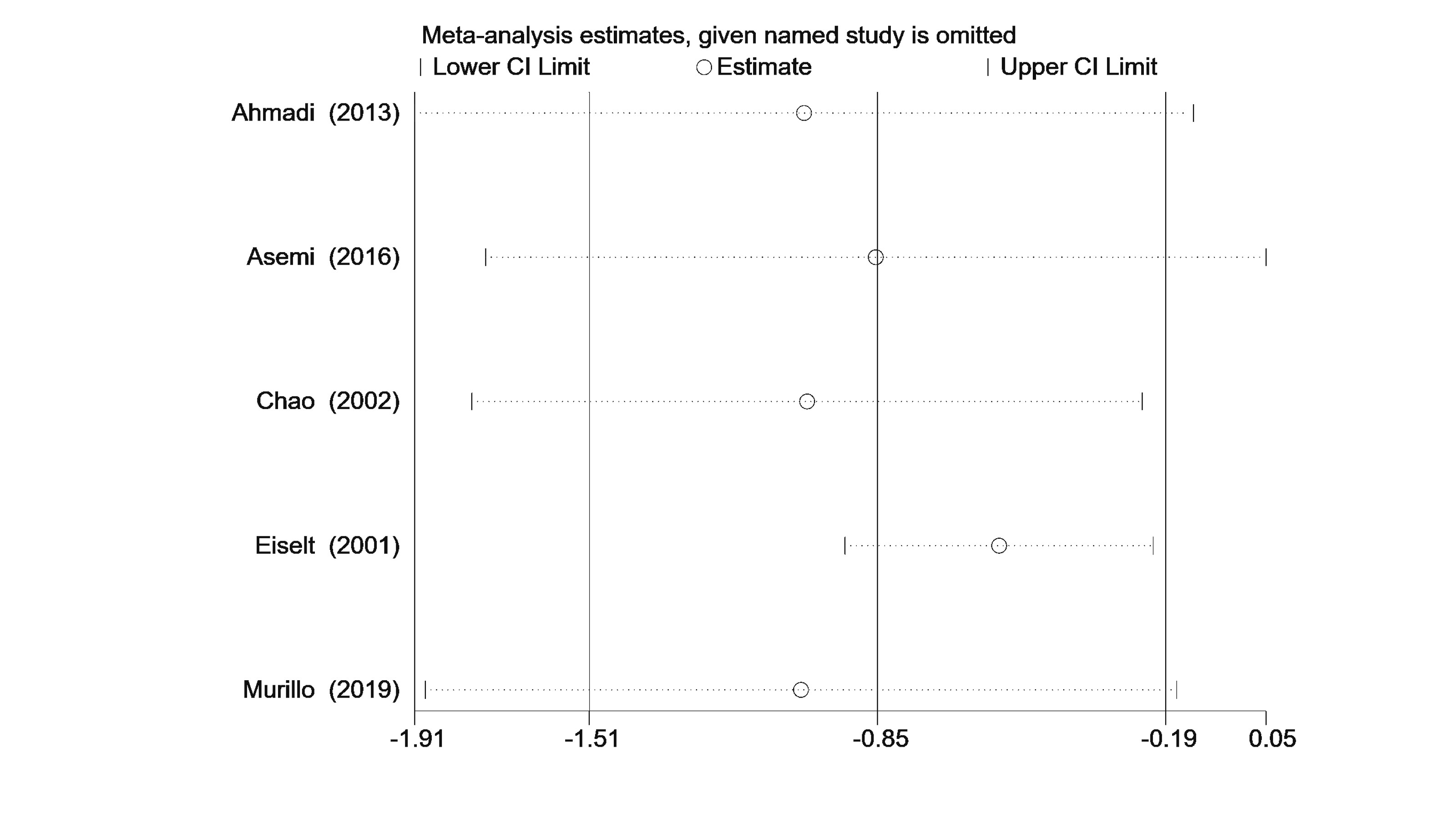

Supplement: Supplementary file 1 [file Data_Sheet_1.zip › Supplementary Material Presentation/Supplementary_Figures/Fig. 4f Sensitivity analysis of multiple antioxidant interventions.jpg]
