# Supplementary material for: Efficacy of antioxidant intervention and exercise intervention for lipid peroxidation in dialysis patients: a meta-analysis
Source: Front Med (Lausanne). 2025 Mar 17;12:1473818. doi: 10.3389/fmed.2025.1473818 (PMC11955636; doi:10.3389/fmed.2025.1473818)
Supplement: Supplementary file 1 [file Data_Sheet_1.zip › Supplementary Material Presentation/Supplementary Appendix A.doc]

**Table 1 Basic information of the single-experiment intervention**

| Study | Country | Additional  Intervention | Intervention time | Duration of dialysis (month) | Age | Outcome indicator (source) (unit) | |
| --- | --- | --- | --- | --- | --- | --- | --- |
|  |  |  |  |  |  | Mean ± SD  Control | Mean ± SD  Experimential |
| Castilla P 2008^[1]^ | Spain | Concentrated red grape juice (50ml/d) | 14days | ≥3 | 33-79 | Ox-LDL(S) (U/L)  126 ± 19 | Ox-LDL(S) (U/L)  43 ± 8 |
| Castilla P 2008^[1]^ | Spain | Vitamin E (800IU/d) | 14days | ≥3 | 33-79 | Ox-LDL(S) (U/L)  126 ± 19 | Ox-LDL(S) (U/L)  91 ± 8 |
| Castilla P 2008^[1]^ | Spain | Concentrated red grape juice (50ml/d) + Vitamin E (800IU/d) | 14days | ≥3 | 33-79 | Ox-LDL(S) (U/L)  126 ± 19 | Ox-LDL(S) (U/L)  84 ± 14 |
| Groussard C 2015^[2]^ | France | Intradialytic training | 3months | >3 | 66.50 ± 4.60 | Ox-LDL(S) (U/L)  39.70 ± 4.60 | Ox-LDL(S) (U/L)  40.6 ± 3.4 |
| Lu L 2007^[3]^ | America | Vitamin E (800IU/d) | 6months | 5-96 | 31.70-86.10 | 4-HNE (nmol/ml)  0.51 ± 0.11 | 4-HNE (nmol/ml)  0.51 ± 0.08 |

[1]	Castilla P., Dávalos A., Teruel J.L., Cerrato F., Fernández-Lucas M., Merino J.L., et al., Comparative effects of dietary supplementation with red grape juice and vitamin E on production of superoxide by circulating neutrophil NADPH oxidase in hemodialysis patients. *The American journal of clinical nutrition* (2008) 87:1053-61.doi:10.1093/ajcn/87.4.1053

[2]	Groussard C., Rouchon-Isnard M., Coutard C., Romain F., Malardé L., Lemoine-Morel S., et al., Beneficial effects of an intradialytic cycling training program in patients with end-stage kidney disease. *Applied physiology, nutrition, and metabolism = Physiologie appliquee, nutrition et metabolisme* (2015) 40:550-6.doi:10.1139/apnm-2014-0357

[3]	Lu L., Erhard P., Salomon R.G., and Weiss M.F., Serum vitamin E and oxidative protein modification in hemodialysis: a randomized clinical trial. *American journal of kidney diseases : the official journal of the National Kidney Foundation* (2007) 50:305-13.doi:10.1053/j.ajkd.2007.05.001
